# Evolutionarily conserved neural signatures involved in sequencing predictions and their relevance for language

**DOI:** 10.1016/j.cobeha.2018.05.002

**Published:** 2018-06

**Authors:** Yukiko Kikuchi, William Sedley, Timothy D Griffiths, Christopher I Petkov

**Affiliations:** 1 Institute of Neuroscience, Newcastle University Medical School, Newcastle Upon Tyne, UK; 2 Centre for Behaviour and Evolution, Newcastle University, Newcastle Upon Tyne, UK; 3 Wellcome Trust Centre for Neuroimaging, University College London, UK; 4 Department of Neurosurgery, University of Iowa, Iowa City, USA

## Abstract

Predicting the occurrence of future events from prior ones is vital for animal perception and cognition. Although how such sequence learning (a form of relational knowledge) relates to particular operations in language remains controversial, recent evidence shows that sequence learning is disrupted in frontal lobe damage associated with aphasia. Also, neural sequencing predictions at different temporal scales resemble those involved in language operations occurring at similar scales. Furthermore, comparative work in humans and monkeys highlights evolutionarily conserved frontal substrates and predictive oscillatory signatures in the temporal lobe processing learned sequences of speech signals. Altogether this evidence supports a *relational knowledge hypothesis of language evolution*, proposing that language processes in humans are functionally integrated with an ancestral neural system for predictive sequence learning.

## Highlights

• How sequence learning relates to language operations remains controversial.
• Empirical evidence in humans and comparative studies clarifies and constrains links.
• Neural oscillatory sequencing resembles language operations in time.
• We extend a *relational knowledge hypothesis of language evolution*.
• Ancestral neural system is integrated with analogous temporal functions in language.

**Current Opinion in Behavioral Sciences** 2018, **21**:145–153

This review comes from a themed issue on **The evolution of language**

Edited by **Christopher Petkov** and **William Marslen-Wilson**

For a complete overview see the Issue and the Editorial

Available online 1st June 2018

**https://doi.org/10.1016/j.cobeha.2018.05.002**

2352-1546/© 2018 The Authors. Published by Elsevier Ltd. This is an open access article under the CC BY license (http://creativecommons.org/licenses/by/4.0/).

## Introduction

The human language faculty is unique in the animal kingdom because it harnesses open-ended combinatorial capabilities operating on a massive semantic store. Language affords humans the capacity to comprehend and to produce structured sentences of speech sounds, visual symbols or signs, with informative content at multiple temporal scales (phonemic, syllabic, syntactic, etc.). There is general agreement that the human language faculty is not monolithic, but has core phonological, semantic and syntactic components (see Friederici, Hagoort and Marslen-Wilson papers in this issue). However, consensus is lacking on which functions are language-specific and which engage cognitive domain-general operations not specific for language [1, 2, 3, 4] (also see Campbell & Tyler in this issue). This issue may be better understood by asking which aspects of human language rely on evolutionarily conserved neurocognitive processes.

In this article, we discuss converging empirical evidence on the neurobiology of sequence learning and natural language. Sequence learning tasks, including those that use Artificial Grammar (AG) learning paradigms, are designed to emulate rule-based dependencies in language across various temporal scales and distances. These tasks do not engage identical processes as those in language, such as syntactic operations on semantic units, but recent work has shown that such sequence learning capabilities, firstly have associations to temporally corresponding language operations in children and adults, secondly are seen to engage parts of the fronto-temporal language network, again for processing at similar temporal scales, and finally form a core part of the impairments seen in aphasic patients with grammatical difficulties. Also, neural oscillations, which reflect the coordination of neuronal populations, are ubiquitous in the brain and are seen to be crucial for segmenting the temporal structure of speech signals and lexical or phrasal dependencies in a sentence. Moreover, comparative work using sequence learning tasks is identifying the evolutionarily conserved processes and neural temporal predictive operations involved, which are seen to reside in regions homologous to those supporting certain speech and language-related processes in humans. On the basis of the combination of this evidence, we extend a *relational knowledge hypothesis* on the origin of language, proposing that certain fronto-temporal language operations are integrated with an evolutionarily conserved system for predictive sequence learning, particularly when processes require neural operations at corresponding temporal scales. Finally, the synopsis highlights empirical pathways for advancing our understanding of the human language system and its likely evolutionary precursors.

## Empirical links between sequence learning and analogous temporal operations in language

**Figure 1:**
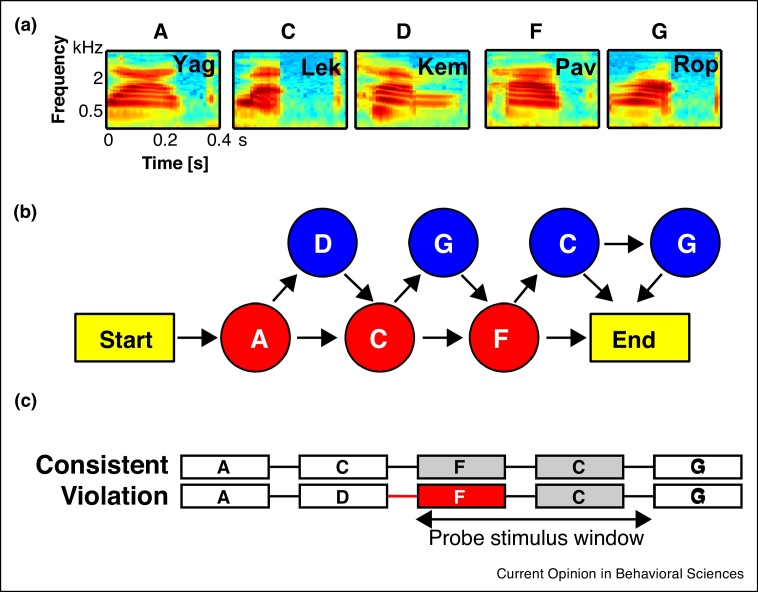
An Artificial Grammar (AG) learning paradigm establishing probabilistic transitions between nonsense words in a sequence. **(a)** Spectrograms of the five nonsense word elements used in the study by Kikuchi *et al.* [71^••^]. **(b)** The AG used was developed by Saffran and colleagues [80], also see [75,81]. It consists of obligatory (red) and optional (blue) nonsense word elements. In the illustration, following any of the arrows from start to end generates a legal ‘consistent’ sequence. **(c)** Example consistent and matching violation sequence pair. The red box highlights the first illegal sound element in the sequence. Neural responses were measured after this illegal transition over a probe stimulus window that contained identical acoustical items as with the matched consistent sequence, which was wholly consistent with the learned AG sequencing relationships.

Rule-based sequence learning paradigms (Figure 1) were originally employed to study human infants and adults [5, 6, 7] and are also used to comparatively test the sequence learning capabilities of nonhuman animals [8,9]. Typically, there is an initial learning phase, via exposure or operant training, where the participants experience exemplary sequences following a specific set of rule-based dependencies; for example, stimulus A can be followed by stimuli C or D with some probability, and D is always followed by C for a sequence including these stimuli to be legal (Figure 1b). Then, in a subsequent testing phase, novel test sequences are presented, which either follow or violate the learned sequencing dependencies. Behavioral or neural responses to consistencies or violations in the sequencing relationships can therefore determine which ordering dependencies humans or other animals can process and the neural substrates involved.

A number of sequence learning abilities now have established links to language in humans, and some of these abilities are known to be evolutionarily conserved in nonhuman animals. Predictive sequence learning is associated with infant and adult language processing [10, 11, 12, 13, 14], and sequencing capabilities are impaired in developmental language disorders, including specific language impairment [15,16] and dyslexia [17]. For example, 7-month-old infants show similar order sensitivity during an artificial grammar learning task as they do with the word order dependencies present in their natural language (Japanese infants can expect the opposite word order from English infants: the equivalent of *Tokyo ni* ‘Tokyo to’ in Japanese is ‘to Tokyo’ in English) [18^••^]. As another example, within a serial reaction time task, the ability of adults to process an artificial grammar with non-adjacent dependencies (an A*X*B paradigm where A and B items are associated with one another across the intervening *X* items) is associated with the speed of reading object-relative rather than subject-relative clauses in natural language, the latter of which are quicker to parse [13]. There is also growing evidence from comparative behavioral work that nonhuman animals such as primates, songbirds and rodents can process adjacent and non-adjacent sequencing dependencies between items in a sequence [19,20^•^,^21^, ^22^, ^23^, ^24^].

Additional empirical evidence for links between sequence processing and related temporal scales of analysis in language comes from patient studies and neurobiological data. Aphasic patients with prefrontal vascular or degenerative pathologies affecting their grammatical abilities are also severely impaired on sequence processing tasks using speech or non-speech sounds [25, 26, 27]. The sequence processing deficits appear to affect simpler predictable adjacent dependencies between two items in a sequence through to more complex sequencing dependencies [28^•^].

Neurobiological studies in healthy humans have shown that processing AG sequences of different forms of complexity engages distinct frontal and temporal brain regions and pathways. Adjacent operations on words in a sentence or analogous operations in AG learning tasks, such as the processing of adjacent dependencies between items, primarily involve the ventral processing stream interconnecting anterior temporal to inferior frontal areas such as the frontal opercular cortex [20^•^,^29^]. By comparison, in humans, more complex non-adjacent or hierarchically organized dependencies during language processing or AG learning tasks additionally engage regions interconnected by the dorsal arcuate fasciculus pathway, including Broca’s area (Brodmann areas 44/45) [30, 31, 32]. We refer the reader elsewhere for details on how the involvement of the frontal system depends on language syntax or sequencing structural complexity [20^•^,^29^].

Recent comparative neuroimaging work in monkeys and humans has identified cross-species correspondences in the frontal operculum for processing adjacent sequencing dependencies [33]. The study also found that the level of involvement of neighboring prefrontal regions involving Brodmann areas 44/45 was minimal in humans but more variable in the monkeys. It is thus possible that BA44/45 in humans has evolved to cope with more complex sequencing dependencies and those required for language [20^•^], or to better integrate different cognitive operations, such as the number of items and their sequencing relationships [34]. However, how the human inferior frontal cortex may have mechanistically differentiated and for which purposes is unknown, requiring further human work at the interface of language and domain general operations complemented by comparative work on temporal dependencies in nonhuman animals.

Humans harness their syntactic and semantic knowledge to build complex meaningful expressions, often creating hierarchical dependencies between words or phrases in a sentence [1]. While certain whale and songbird songs contain phrases and simpler hierarchical organization of song units [35], whether any nonhuman animal can learn to process ‘language-like’ hierarchically organized relationships remains controversial [36]. On the other hand, nonhuman primates, for instance, can organize complex motor sequences [37], evaluate social knowledge based on a rich hierarchy of social relations [38], and their prefrontal cortex richly and dynamically encodes cognitive behavior over time [39]. Thus, the full extent of nonhuman animal sequence processing capabilities, the phylogenetic pattern of complexity in those capabilities, which types of hierarchical operations nonhuman animals are able to learn and the correspondences that can be made to language-related operations in humans remain outstanding questions.

## The need to anticipate: predictive coding of environmental events and cross-frequency oscillatory coupling

Intrinsic neural oscillations are ubiquitous in the brain and can be categorized into different oscillatory frequency bands reflecting different neurobiological functions. For instance, memory-related operations [40] and attentional sampling [41] are associated with low frequency neural oscillations, such as those in the theta frequency range (∼4–8 Hz). Populations of neurons can also entrain their oscillations to rhythmic sensory input, both reactively and preemptively [42, 43, 44]. The latter is thought to constitute a form of sensory prediction manifest in hierarchically higher brain areas, as we consider.

The predictive coding framework posits that higher level brain areas send predictions to hierarchically earlier sensory areas [45], in the form of beta frequency oscillations (∼15–30 Hz) [46]. These predictions are assessed alongside ascending sensory input, and any discrepancies generate a prediction error signal [47, 48, 49], which is relayed to higher level areas in the form of gamma band activity (>30 Hz). There can also be cross-frequency coupling, such as the phase of low frequency signals coordinating with high frequency signal amplitude, known as phase-amplitude coupling (PAC). PAC is a signature of information transfer between neural populations within and between spatially segregated brain regions [50,51]. Neural oscillations and oscillatory coupling are impaired in many neurological and psychiatric disorders [52], such as over-coupling in Parkinson’s patients in the beta and high-gamma bands [53] or under-coupling in autism or schizophrenia in the alpha/gamma band [54].

The research community now has a detailed understanding of how rhythmic activity entrains the brain at particular oscillatory frequencies. We also better understand how expected or unexpected (oddball) sounds elicit prediction errors in the brain [55, 56, 57]. Much less is known about how sequence learning affects neural oscillations and how these relate to speech and language processes.

## Neural oscillatory responses to speech

Speech has temporal regularities at multiple scales (e.g. phonemic, syllabic, and phrasal rates) [58,59]. For example, syllabic content occurs in an approximately theta frequency cycle (4–8 Hz). This rhythm is consistent across languages [60] and is also present in primate vocalizations [61]. In human auditory cortex, neural oscillations can entrain to the syllabic and phonemic content in speech [59,62]. For example, phase entrainment of speech signals at the syllabic rate is thought to be a core process for perceptual segmentation of continuous speech into its constituent parts [63,64]. A prominent neurobiological model [59] postulates that theta phase entrainment to the syllabic rate couples with high-frequency gamma amplitude (>30 Hz), resulting in theta-gamma phase-amplitude coupling as measured in local field potential, EEG or MEG signals.

Neural oscillatory responses in temporal cortex are modulated within different oscillatory frequency bands during phonotactic segmentation [65], by between-word phrases [66^••^,^67^] and as a function of working memory demands in sentence comprehension [68]. As another example, in Mandarin speakers, segmenting Chinese phrases that occur at a lower rate (∼2 Hz) results in modulation of low-frequency oscillations in fronto-temporal regions that phase-lock to the perceived phrase structure [66^••^]. Such low-frequency neural tracking of phrasal structure may further modulate higher frequency neural oscillations such as those in the gamma band [59]. Another intracranial recording study in humans using natural sentences shows that as words within a phrase are being processed there is an accumulation of frontal neural activity in the gamma range [67]. Once a phrase boundary occurs there is a drop of gamma activity, possibly indicative of a change in representation from individual words to a phrase. Furthermore, recent patient work suggests that the primary deficit in prefrontal cortex atrophy is not the formation of predictions per se, but that speech predictions are overly precise and inflexible [69^••^]. These disrupted predictions are linked to increased pre-stimulus beta band oscillatory activity in the patients that can be detrimental for speech perception. Thereby, predictive neural operations at various temporal scales feature prominently not only in processing sequences of environmental events, but also for processing speech and language.

## Conserved neural oscillatory coupling and sequencing predictions in human and monkey auditory cortex

Two recent studies show that speech and sequencing predictions in auditory cortex are evolutionarily conserved between humans and monkeys [70,71^••^]. Both studies found the morphology of oscillatory coupling to speech signals to be remarkably similar, as we consider here.

**Figure 2:**
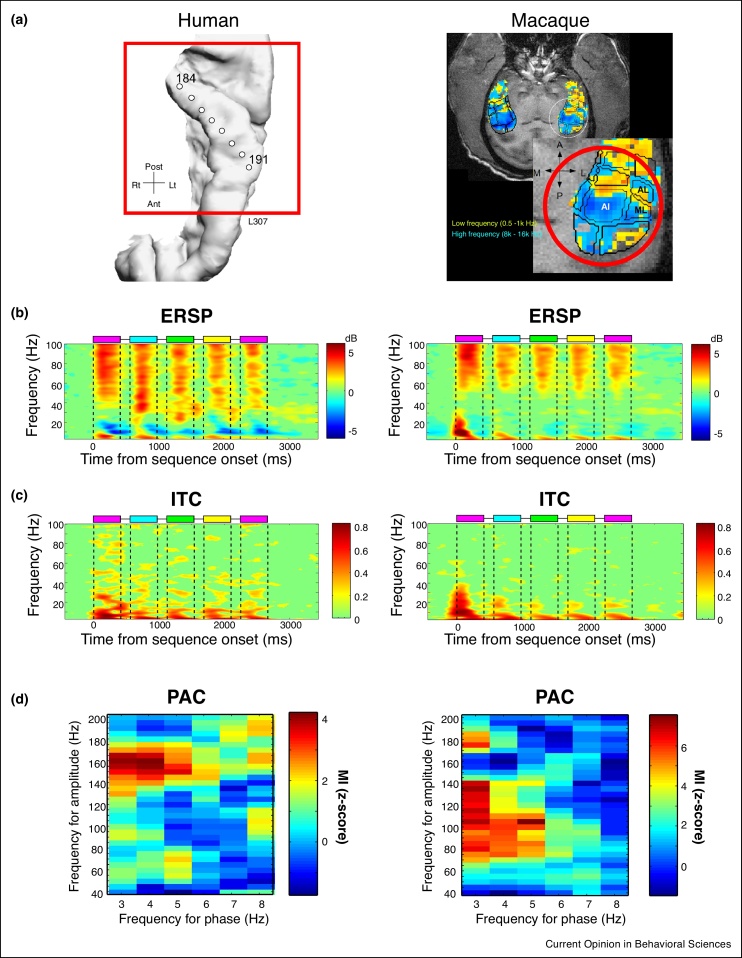
Conserved neural signatures in human (left column) and monkey (right column) auditory cortex in response to sequences of nonsense words. **(a)** Recording sites in the human Heschl’s gyrus (left panel) and macaque auditory cortex (right panel). The macaque structural MRI image on the right shows an axial MRI slice looking down on the supratemporal plane overlayed with a functionally defined auditory tonotopic map. **(b)** Time–frequency responses to each of the sounds in the sequence, shown as power changes (event-related spectral perturbation, ERSP) in the recorded local field potentials (LFPs) from human (left panel) and monkey (right panel) auditory cortex. Colored boxes on the top of the plots identify the time of occurrence of the different nonsense words. Note the prominent high gamma power responses to each of the speech sounds in a sequence. **(c)** Plots of the inter-trial phase coherence (ITC) across the frequency bands and in response to the sequences of sounds. These show phase alignment at particular frequency bands (such as theta; 4–8 Hz). **(d)** Exemplary phase-amplitude coupling (PAC) in response to the nonsense words. The modulation index (MI) values show the strength of PAC for each combination of low frequency phase (*x*-axis) and high frequency amplitude (*y*-axis).

Zoefel and colleagues recorded from monkey primary auditory cortex (A1) neurons and report theta-gamma coupling in response to natural speech [70], similar to speech responses in human EEG signals [72]. Kikuchi and colleagues [71^••^] recorded from primary and adjacent auditory cortical regions in monkeys in response to sequences of speech sounds, comparing the neural responses to these signals in monkeys with those obtained in humans from intracranial depth electrode recordings of Heschl’s gyrus. The study showed similar theta-gamma coupling in the human and monkey auditory cortex in response to the speech sounds (Figure 2), supporting the notion of evolutionarily conserved neural oscillatory processes for speech sounds in auditory cortex.

The study by Kikuchi and colleagues also assessed the processing of adjacent sequencing relationships, using an AG learning paradigm that regulates the predictability of the between word transitions [71^••^]. After exposing the humans and monkeys to sequences that establish the AG sequencing dependencies, they tested the two species with novel sequences that were consistent with or in violation of the learned AG sequencing relationships. In both species, they saw that theta-gamma coupling, a sequencing prediction error signal, was increased by an illegal sequencing transition in the violation sequences. They also saw that in a different subset of neurons the theta-gamma coupling strength was increased by the legal predicted sequencing relationships present in the sequences consistent with the AG.

**Figure 3:**
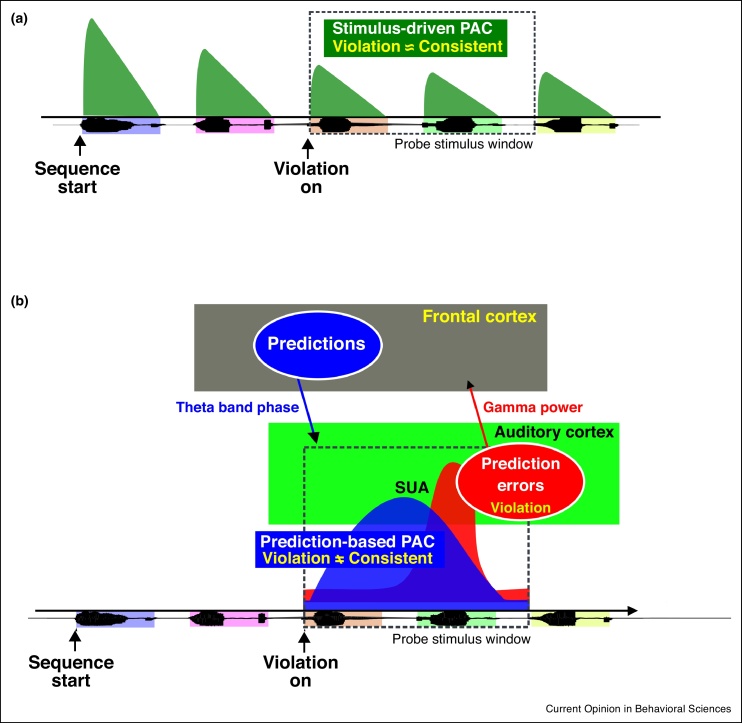
A physiologically informed model of sequencing predictions in time. This physiological model is based in part on the results of the study by Kikuchi and colleagues [71^••^]. **(a)** Speech signals, as complex sounds, entrain to low-frequency phase that further coordinates with high frequency amplitude, resulting in phase-amplitude coupling (PAC). **(b)** After exposure to structured sequencing relationships, different neural signals (LFP, SUA, oscillatory coupling) show sequencing context-dependent response modulations, lagging sound onset. Prediction signals, reflected in PAC and likely emanating from hierarchically higher brain areas such as frontal cortex or the hippocampus, occur when the ordering relationships are consistent with the learned sequence ordering relationships. These influence auditory cortical neurons prior to concomitant effects being seen in local field potential power. This prediction signal accumulates and is modulated later in time (∼600 ms) when a sequencing violation occurs (a prediction error), evident as high-gamma power predominantly responding to the violation sequences, see [71^••^].

With monkeys as a model system in which a substantial number of single neuron responses can be recorded, the authors were able to link the observed neural oscillatory responses to local single neuron activity. This is illustrated in Figure 3, which presents a physiological model of predictive sequencing operations in auditory cortex. Here it can be seen that stimulus-driven theta-gamma coupling occurs in response to each of the speech sounds in the sequence (green in Figure 3). However, sequencing prediction and prediction error signals are distinct from stimulus driven effects. Namely, if a correctly predicted transition occurs, a predictive signal (blue) is seen to accumulate later in a subset of neural responses (∼500 ms). If, however, a sequencing violation has occurred, this manifests at an even later time (∼600 ms) as modulation of theta-gamma coupling in another neural subpopulation (red). This relatively late neural signal associated with sequencing prediction errors matches a late event related potential seen in human and macaque EEG [73,74]. Also, the neurophysiological prediction error signal from auditory cortex occurs at a behaviorally meaningful time, at the approximate time that macaque monkey eye tracking data shows that they notice specific sequence order violations [75]. The later neural response latency in relation to the relatively earlier accumulation of predictive signals may stem from the need to accumulate information to assess sensory input in relation to predictive signals likely emanating from other sites interacting with auditory cortex. Thus, distinct sequencing prediction effects segregate in both space and time, with theta driven phase-amplitude coupling coordinating in tandem with local single neuron responses, prior to effects on other neural responses (Figure 3).

These neural results on sequence processing are generally consistent with the predictive coding framework [71^••^]. We further postulate that low-frequency theta oscillations may be a feedback prediction signal from inferior frontal cortex [33] and/or the hippocampal memory system [76] that influences auditory cortical neuronal responses involved in segmenting complex signals, such as speech. The high-gamma responses related to sequencing violations appear to be a sequencing prediction error signal that is relayed forward from auditory cortex to hierarchically higher level brain areas [77]. Feedback signals may enhance low-frequency phase in auditory cortex, strengthening the gamma prediction error signal as a function of the learned sequencing relationships.

In summary, auditory cortex neural responses in humans and monkeys show a signature of learned sequencing dependencies, which is seen to be remarkably similar across the species and is now linked to single neuron responses in monkeys as a model system. Further comparative work is needed to identify the feedforward and feedback processes involved in sequence learning and how these predictive neural processes compare across the species and with temporally aligned language-specific processes that can be studied in humans.

## The relational knowledge hypothesis of language origins

Wilson and Petkov motivated a *relational knowledge hypothesis of language evolution* [78], developed from observations of primate sequence learning behavior and how monkeys apply their social knowledge during natural vocal interactions [38]. We extend this hypothesis here with the neurobiological observations that were considered above.

Sequence learning is a form of relational knowledge [79], where temporal dependencies are established via learning at the appropriate temporal granularity. After learning, the brain evaluates incoming sequences of sensory events in relation to expectations from previously learned sequencing dependencies in the form of feedback from hierarchically higher frontal and other sites. When predictions for subsequent sequences cannot be supported, a sequencing prediction error results and updates synaptic weights that are fed-forward throughout the network to update future predictions. Differential aspects of the neurocognitive system, including broader aspects of inferior frontal cortex, are likely engaged as a function of the complexity of the temporal dependencies [20^•^], as is also seen for language syntactic operations [29].

## Conclusions

Language-critical processes in humans appear to be functionally integrated with an ancestral neural system supporting relational knowledge, such as sequence learning. The extent to which this or any other domain general neural system can be segregated from the one supporting language is an active area of research aiming to clarify the neural specializations for language. It remains possible that two separate systems exist side-by-side in humans, by way of evolutionary duplication and differentiation of general processes for language. Even so, it follows that at some levels a shared process can identify the generic neural mechanisms involved, aspects of which could be modelled in nonhuman animals at the circuit, cell and molecular levels if the process is also shown to be evolutionarily conserved. The relevance to language notwithstanding, understanding the impact of serial order on the brain and behavior remains an important endeavor. Thus future studies could seek to clarify the laminar and inter-regional feedforward and feedback neural dynamics involved in predicting environmental events at different temporal scales, perturbing the system as necessary to establish causal relationships.

## Funding

This work was supported by Wellcome Trust [grant numbers WT091681MA received by TDG and WT092606AIA received by CIP and YK]; U.K. Biotechnology and Biological Sciences Research Council [grant number BB/J009849/1 received by CIP and YK, joint with Quoc Vuong]; NPO NeuroCreative Lab award received by YK; National Institutes of Health intramural contract received by CIP and YK; NIH extramural grant; the National Institutes of Health [grant number R01-DC04290 received by Matthew Howard III, joint with TDG and CIP].

## Conflict of interest statement

Nothing declared.

## References and recommended reading

Papers of particular interest, published within the period of review, have been highlighted as

• of special interest
•• of outstanding interest
